# Altered Gut Microbiota Composition Associated with Eczema in Infants

**DOI:** 10.1371/journal.pone.0166026

**Published:** 2016-11-03

**Authors:** Huajun Zheng, Hong Liang, Yuezhu Wang, Maohua Miao, Tao Shi, Fen Yang, Enuo Liu, Wei Yuan, Zai-Si Ji, De-Kun Li

**Affiliations:** 1 Key Laboratory of Reproduction Regulation of NPFPC, SIPPR, IRD, Fudan University, Shanghai 200032, China; 2 Shanghai-MOST Key Laboratory of Health and Disease Genomics, Chinese National Human Genome Center at Shanghai, Shanghai 201203, China; 3 Division of Research, Kaiser Foundation Research Institute, 2000 Broadway, Oakland, CA 94612, United States of America; Columbia University, UNITED STATES

## Abstract

Eczema is frequently the first manifestation of an atopic diathesis and alteration in the diversity of gut microbiota has been reported in infants with eczema. To identify specific bacterial communities associated with eczema, we conducted a case-control study of 50 infants with eczema (cases) and 51 healthy infants (controls). We performed high-throughput sequencing for V3–V4 hypervariable regions of the 16S rRNA genes from the gut fecal material. A total of 12,386 OTUs (operational taxonomic units) at a 97% similarity level were obtained from the two groups, and we observed a difference in taxa abundance, but not the taxonomic composition, of gut microbiota between the two groups. We identified four genera enriched in healthy infants: *Bifidobacterium*, *Megasphaera*, *Haemophilus* and *Streptococcus*; and five genera enriched in infants with eczema: *Escherichia/Shigella*, *Veillonella*, *Faecalibacterium*, *Lachnospiraceae incertae sedis* and *Clostridium XlVa*. Several species, such as *Faecalibacterium prausnitzii* and *Ruminococcus gnavus*, that are known to be associated with atopy or inflammation, were found to be significantly enriched in infants with eczema. Higher abundance of *Akkermansia muciniphila* in eczematous infants might reduce the integrity of intestinal barrier function and therefore increase the risk of developing eczema. On the other hand, *Bacteroides fragilis* and *Streptococcus salivarius*, which are known for their anti-inflammatory properties, were less abundant in infants with eczema. The observed differences in genera and species between cases and controls in this study may provide insight into the link between the microbiome and eczema risk.

## Introduction

Eczema is a chronic inflammatory disorder of the skin, which commonly starts during infancy. Its prevalence has been increasing worldwide, and it is more prevalent in affluent societies. It afflicts children mainly in the first year of life (60%) and affects up to 30% of infants [1, 2], while the condition may persist into adulthood. Eczema is frequently the first manifestation of an atopic diathesis [3]. Eczema is one of the strongest predictors of allergic diseases [3–5]. Up to 80% of children with eczema will eventually develop allergic rhinitis or asthma within five years of diagnosis. Eczema could be divided into "atopic eczema" (IgE-mediated) and "non-atopic eczema” according to its mechanism [6].

After the term “probiotic” was defined by Lilly DM in 1965 [7], several probiotics have been used for clinical treatment or prevention of eczema by reducing the IgE level; most of them belong to *Lactobacillus* and *Bifidobacterium* [8]. The questions of how gut microbial communities and immune systems co-evolve during our lifespans, especially in the early life stages and how components of the microbiota affect the immune system remain largely unanswered [9]. It has become increasingly clear that the gut microbiota plays an important role in the regulation of innate and adaptive immunity, and has been implicated in the development of allergic diseases [10]. Members of the genus *Bifidobacterium* dominate the large-bowel bacterial community of human infants during the first months of life [6].

The diversity of the gut microbiota was reported to be significantly reduced in infants with eczema [11, 12]. Most strikingly, some probiotics belonging to *Bifidobacterium* and *Lactobacillus* are present at a significantly greater abundance in non-eczematous infants at several time points (1–3 and 12 months, two and five years) [10, 13, 14]. On the contrary, pathogenic bacteria belonging to *Enterococcus* and *Shigella* had a greater abundance in eczematous infants during the early stages of infancy [13, 14].

We conducted this study to compare the structures of gut commensal bacteria between healthy infants and infants who had experienced eczema during the first year of their life through a stool sample collected at 12–13 months of age, and to identify specific bacterial communities that may be associated with eczema. We performed high-throughput sequencing for V3–V4 hypervariable regions of the 16S rRNA gene from gut fecal material to characterize and compare the difference in the gut microbiota between healthy and eczematous infants.

## Methods

### Ethics Statement

The study was reviewed and approved by the ethics committee board of Shanghai Institute of Planned Parenthood Research (IRB00008297). All participants gave their written informed consent before participation in the study.

### Sample collection

We conducted a case-control study to examine the association between microbiome and the risk of eczema among infants. Study subjects were selected from a birth cohort, in which 1,400 pregnant women were recruited in their first trimester in the Maternity and Child Care Hospital, Minhang District of Shanghai. Of the followed women, 1,048 delivered live births. We conducted home visits for all live-born infants at 12 months after obtaining their mothers’ consent. During the visit, the parents were asked to report their children’s history of physician-diagnosed eczema since birth using a structured questionnaire. Among them, 150 infants were invited to participate in a study examining the microbiota of infants. A total of 101 mothers agreed to participate and infants’ stool samples were collected at 12–13 months of age using a self-collection kit following a standard collection procedure. Among the 101 fecal samples, 51 samples were from case infants who had developed eczema within the first 12 months, while 50 were from healthy control infants who had not developed eczema during the first year of life.

### DNA extraction

DNA was extracted from 300 mg of feces using a QIAamp DNA stool mini kit (Qiagen, Hilden, Germany) according to the manufacturer’s instructions. The amount of DNA was determined using a Qubit^®^ 2.0 Fluorometer (Life Technologies, USA); the integrity and size were checked by 1.5% agarose gel electrophoresis containing 0.5 mg/ml ethidium bromide. All DNA was stored at -20°C until further analysis.

### PCR and pyrosequencing

The bacterial genomic DNA was amplified with 343F (5’- TACGGRAGGCAGCAG-3’) and 798R (5’- AGGGTATCTAATCCT -3’) primers specific for the V3-V4 hypervariable regions of the 16S rRNA gene [15]. Barcodes that allowed sample multiplexing during pyrosequencing were incorporated between the 454 FLX Titanium adapter and the 5’ end of the forward primer. The thermocycling steps were as follows: 95°C for 5 min, followed by 20 cycles at 95°C for 30 sec, 55°C for 30 sec, 72°C for 1 min and a final extension step at 72°C for 10 min.

Each PCR reaction was performed in a 50 μl system, and the products were extracted with the QIAquick gel extraction kit (Qiagen) and quantified on a NanoDrop 2000C spectrophotometer and Qubit^®^ 2.0 Fluorometer (Life technologies).

The samples were pooled in an equal amount and sequenced on a Roche 454 GS FLX+ System (Roche, Basel, Switzerland) according to the manufacturer’s recommendations. The sequencing was started from the the 454 FLX Titanium adapter.

### Bioinformatics and statistical analysis

Raw pyrosequencing reads obtained from the sequencer were first treated using the 454 SOP of Mothur (version 1.32.0) [16], in a process including denoising and removal of chimera and contaminants. Next, sequences shorter than 350 nucleotides, those with homopolymers longer than eight nucleotides, those with average sequence quality values below 25, any reads containing ambiguous base calls or those that had even one incorrect primer sequence were filtered out.

The high-quality sequences were assigned to samples according to barcodes. After the removal of primers, operational taxonomic units (OTUs) were calculated by comparing to the SILVA [17] reference database (V119) using Mothur; the data for 101 samples were normalized using command "sub.sample”. OTUs that reached a 97% nucleotide similarity level were used for community richness and diversity analysis (Shannon, Simpson, ACE, Chao1 and Good’s coverage). The Good’s coverage of 101 samples was 96.81%±2.0%. Principal coordinate analysis (PCoA), Shannon-Weaver diversity index and weighted uniFrac distance metrics analysis were performed and Venn diagrams derived, using OTUs for each sample obtained through the Mothur program.

The online software RDP classifier [18] was used to assign sequences to phylogenetic taxonomy based on the Ribosomal Database Project [19]; the sequences were assigned to the hierarchical taxa under the condition of a bootstrap cutoff of 80%. The features (OTUs, genera, etc.), that were differentially abundant between the two groups were determined by the Metastats [20] program; the features with a q-value <1E-5 (an individual measurement of the false discovery rate) were considered as significant (differentially abundant). The software Genesis (version 1.7.6) [21] was used to draw heatmaps based on the Metastats-derived results of features. LDA Effect Size (LEfSe) [22] was used for biomarker discovery. The Silva database (version 119, SSU_Ref) was used to identify species through BlastN, and the parameters were set as identity ≥99% and alignment ≥97%. Finally, the best hit was chosen to be reconfirmed on the NCBI’s nt database.

### Sequence read accession number

The authors confirm that all data underlying the findings are fully available without restriction. The sequence data from this study have been deposited in the GenBank Sequence Read Archive under accession number SRS1054294.

## Results

### Characteristics of Samples with/without eczema

A total of 972,520 high quality sequences were generated from 50 eczema cases and 51 controls whose characteristics are presented in Table 1. An average of 9,629 (range: 6,134 to 13,087) sequences per barcoded sample was recovered for downstream analysis. A total of 500,553 sequences were obtained from healthy infants for phylogenetic analysis, while 471,967 sequences were obtained from infants with eczema.

**Table 1:** Descriptive data of infants in the study.

Parameters	Health control (n = 51)	Eczema (n = 50)
**Breast-fed/formula-fed**	25/20	20/27
**Cesarean/ vaginal delivery**	17/25	19/25
**Obese/normal weight**	8/39	7/39

Since the sample with the fewest sequences had 6,134 reads, we normalized each sample to 6,134 reads using command "sub.sample” of Mothur. After data normalization, a total of 12,386 OTUs at a 97% similarity level were obtained from the two groups, comprising 7,418 species level OTUs in healthy control infants and 8,566 OTUs in infants with eczema. The Good’s coverage was estimated to be 98.82% for healthy controls and 98.69% for eczematous infants, indicating a sufficient sequencing depth for an eczema-associated fecal microbiota investigation in infants. This was also supported by the rarefaction curve of 101 samples, which tended to be asymptote after the rarest OTUs (only one observation) were removed according to the former method [23], suggesting that the the most shared species have been obtained.

Neither the bacterial richness(indicated by the ACE and Chao1 values) nor diversity (indicated by the Shannon and Simpson values) was significantly different between the two groups (Table 2, S1 Table, S1 Fig, p-values = 0.56, 0.42, 0.45 and 0.86 for ACE, Chao1, Shannon and Simpson, respectively). The phylogenetic diversity between the two groups was not statistically significant. Accordingly, examining β-diversity by either AMOVA (analysis of molecular variance, Fs = 1.02094, p-value = 0.293) or P-test (Parsimony method, p-value>0.5) showed similar bacterial profiles between the two groups. These observations were also supported by UPGMA algorithm analysis (S2 Fig) and principal coordinates analysis (PCoA) as assessed using the weighted UniFrac metric (S3 Fig).

**Table 2:** Species richness and diversity estimates obtained at genetic distances of 3%.

			Richness		Diversity	
Sample	OTUs	Good's Coverage	ACE	Chao	Shannon	Simpson
**Health control**	**9276**	**0.99083**	**19564.24075**	**15951.94714**	**4.656115**	**0.041557**
**Eczema**	**10409**	**0.98997**	**21352.29745**	**17298.92066**	**4.709316**	**0.047832**

To compare the microbiota composition of two groups, a Venn diagram of OTUs at a 97% similarity level was generated (S4 Fig). Although the results revealed 4,968 unique OTUs in infants with eczema (4.3% of total normalized sequences) and 3,820 unique OTUs in infants without eczema (5.4% of total normalized sequences), no statistically significant differences were revealed between the two groups, likely due to the low proportion of these OTUs.

Through the rank abundance curves of the two groups, we revealed that the majority of the abundance came from a few OTUs (S5 Fig). From the combined OTUs of the two groups, approximately 0.8% of OTUs (top 100 OTUs) accounted for 83.60% of the total normalized sequence in healthy infants and 82.84% in infants with eczema.

### Microbiota between healthy infants and infants with eczema

**Fig 1:**
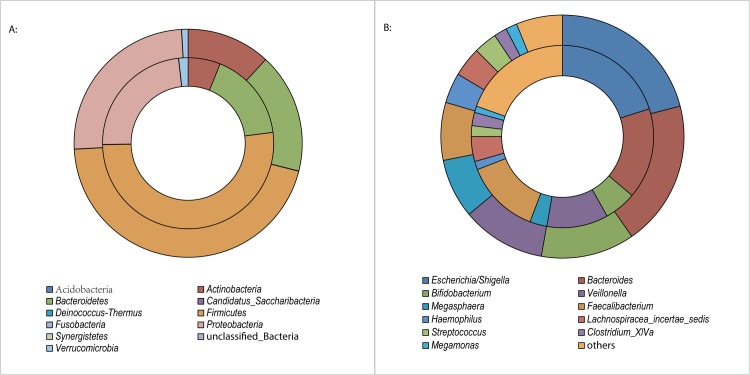
Relative read abundance of different bacterial above a cutoff value of 3%. A: phylum levels; B: genus level. The outer circle represents gut microbiota of healthy infants, and the inner circle represents gut microbiota of eczematous infants.

A total of 10 phyla were found, and the majority of the sequences were assigned to four bacterial phyla (*Firmicutes*, *Proteobacteria*, *Bacteriodetes* and *Actinobacteria*) (Fig 1A). These predominant phyla accounted for 99.00% and 98.13% of the total sequences from healthy infants and infants with eczema, respectively. Additionally, rare phyla, including *Verrucomicrobia*, *Fusobacteria*, *Candidatus Saccharibacter*, *Deinococcus-Thermus*, *Synergistetes* and *Acidobacteria* were present at much lower proportions.

At the family level, 83 families were identified. Two groups shared 57 families. The healthy and eczematous groups had 14 and 12 unique families, respectively. The top ten shared families, which accounted for more than 92.7% of the total sequences, were *Enterobacteriaceae*, *Ruminococcaceae*, *Veillonellaceae*, *Bacteroidaceae*, *Lachnospiraceae*, *Bifidobacteriaceae*, *Verrucomicrobiaceae*, *Streptococcaceae*, *Pasteurellaceae* and *Lactobacillaceae*.

In total, the sequences from fecal microbiota represented 170 bacterial genera, with 143 genera in infants with eczema and 136 genera in healthy infants. The 11 most abundant genera (>1% of total DNA sequences in both two groups) (Fig 1B) accounted for 80.4% and 78.1% of total sequences from infants with eczema and healthy infants, respectively. These were *Escherichia/Shigella*, *Bacteroides*, *Bifidobacterium*, *Veillonella*, *Faecalibacterium*, *Megasphaera*, *Haemophilus*, *Lachnospiraceae incertae sedis*, *Streptococcus*, *Clostridium XlVa* and *Megamonas*. The genera *Bifidobacterium* and *Bacteroides* belonged to the phylum *Actinobacteria and Bacteroidetes*, respectively; and *Escherichia/Shigella* and *Haemophilus* were members of the phylum *Proteobacteria*; the other seven genera were classified to the phylum *Firmicutes*.

### Microbiota differences between healthy infants and infants with eczema

To investigate the difference in the taxa abundance of fecal microbiota between healthy infants and infants with eczema, Metastats was used to identify the differentially abundant phylotypes (taxonomical profiles) including phylum, family and genus (Table 3, S2 and S3 Tables). No significant differences were found between the two groups at the phyla level (p-value = 0.85). Out of the 83 families in the gut microbiota, the ratio of 24 families in the two groups was significantly different (q-value<1E-5), including the above-mentioned top ten shared families with the exception of *Enterobacteriaceae* and *Bacteroidaceae*.

**Table 3:** Abundant species in the gut of infants with and without eczema.

Phylum	genus	species	eczema	health
*Actinobacteria*	*Bifidobacterium*	*Bifidobacterium bifidum*		*
		*Bifidobacterium longum*		*
*Bacteroidetes*	*Bacteroides*	*Bacteroides clarus*	*	
		*Bacterioides plebeius*	*	
		*Bacteroides fragilis*		*
	*Parabacteroides*	*Parabacteroides merdae*	*	
	*Prevotella*	*Prevotella buccae*	*	
*Firmicutes*	*Streptococcus*	*Streptococcus salivarius*		*
	*Blautia*	*Ruminococcus gnavus*	*	
	*Faecalibacterium*	*Faecalibacterium prausnitzii*	*	
	*Subdoligranulum*	*Gemmiger formicilis*	*	
	*Incertae Sedis*	*Eubacterium biforme*		*
*Verrucomicrobia*	*Akkermansia*	*Akkermansia muciniphila*	*	

"*" enriched

**Fig 2:**
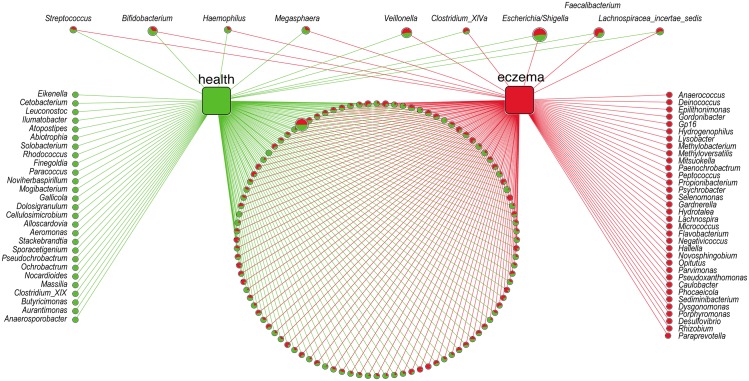
Networks of all the 170 bacteria genera revealed in two groups. Green circle: the unique genera of healthy infants’gut; red circle: the unique genera of eczematous infants. In pie charts, green represents the proportion of this genus in healthy infants and red represents the proportion in eczematous infants. On the top, the nine genera was the most abundance genera (>1% of total DNA sequences in both two groups); the left four genera was enriched in healthy infants, the right five genera was enriched in eczematous infants (q-value<1E-5)). Circle size represented the reads numbers. In the bottom, the pie charts presented all the other bacteria genera common in both groups.

At the genus level, the abundance of 50 genera was identified as being different (q-value<1E-5) between the two groups, including nine out of the 11 most abundant genera (except the genus *Megamonas* and *Bacteroides*) and 41 less abundant genera (Fig 2 and S3 Table). Among the nine most abundant genera, four of them, *Bifidobacterium*, *Megasphaera*, *Haemophilus* and *Streptococcus*, were enriched (q-value<1E-5) in healthy infants; the other five genera, *Escherichia/Shigella*, *Veillonella*, *Faecalibacterium*, *Lachnospiraceae incertae sedis* and *Clostridium XlVa*, were enriched (q-value<1E-5) in infants with eczema. There were 27 and 34 unique genera in the microbiota of healthy infants and infants with eczema, respectively (Fig 2). Despite being unique, the proportion of all unique genera were lower than 0.01%, showing no significant difference between healthy infants and infants with eczema (q-value = 0.01). In infants with eczema, four unique genera were enriched, *Desulfovibrio*, *Paraprevotella*, *Porphyromonas* and *Rhizobium*; while in the healthy infants, no unique genera was enriched.

At the OTUs level, the abundance of 324 OTUs showed a significant difference (q-value<1E-5) between the two groups. In infants with eczema, 170 OTUs were enriched versus 154 in healthy infants. Here, we focused solely on the abundant OTUs (proportion>0.1%), and 18 of them could be assigned into 13 species based on the Silva and NCBI databases (Table 3). Among them, two species belonged to the phylum *Actinobacteria*, five belonged to *Bacteroidetes*, five belonged to *Firmicutes* and one belonged to *Verrucomicrobia*.

**Fig 3:**
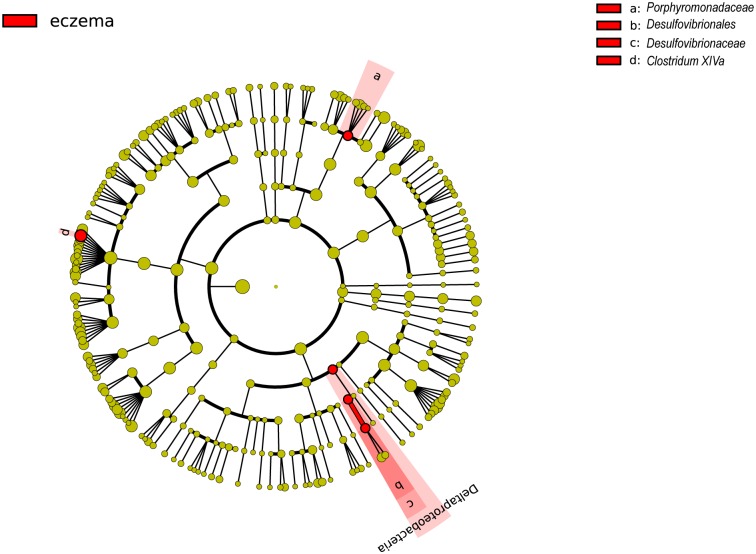
Cladogram of biomarkers for eczematous group. The red circle represented biomarkers and yellow circles represented non-discriminating taxa. Concentric rings from outside to inside were genus, family, order, class and phylum.

To distinguish eczematous infants from healthy infants by intestinal microbiota, we used the program LEfSe to identify potentially differentiating biomarkers. Across all taxonomic levels, only four biomarkers associated with eczema were found, (Fig 3), including the families *Porphyromonadaceae* and *Desulfovibrionaceae* and genus *Clostridium XlVa*. This was consistent with the Metastats-derived results (S2 and S3 Tables), which identified three biomarkers that were abundant in infants with eczema.

Regardless of the mode of delivery (vaginal or caesarean) or type of feeding (infant formula or breast-fed), the following genera were consistently abundant in infants with eczema: *Clostridium XlVa*, *Flavonifractor*, *Gemmiger* and *Lachnospiraceae incertae sedis* (Table 4). The genus *Clostridium XlVa* was identified as a biomarker for eczema (Fig 3). Meanwhile, three genera, *Acidaminococcus*, *Clostridium XI* and *Haemophilus* were highly enriched in healthy infants. These results are consistent with previous studies that reported increased abundance of *Flavonifractor* and decreased *Clostridium XI* in fecal microbiota of infants with a food allergy [24], and increased abundance of the genus *Gemmiger* in obese human gut [25]; and the abundance of the genus *Clostridium XlVa* was usually increased in eczematous infants [26].

**Table 4:** Abundant genus in the gut of infants with and without eczema not affected by mode of delivery or type of feeding.

enrichment	Phylum	Genus	fold_change	Percentage
in health	*Firmicutes*	*Acidaminococcus*	680.01	0.62%
	*Firmicutes*	*Clostridium XI*	3.39	1.20%
	*Proteobacteria*	*Haemophilus*	2.53	3.35%
in eczema	*Firmicutes*	*Clostridium XlVa*	2.02	2.35%
	*Firmicutes*	*Flavonifractor*	3.12	1.68%
	*Firmicutes*	*Gemmiger*	59.82	0.71%
	*Firmicutes*	*Lachnospiracea incertae sedis*	1.48	4.41%

## Discussion

Microbial composition analysis using several bioinformatics methods revealed that, though the taxonomic composition of the intestinal microbiota showed only slight difference between cases and controls, the abundance of OTUs in infants with eczema was significantly different from that of healthy infants.

The family *Enterobacteriaceae* is usually abundant in infants with eczema [14, 27]. In this study, the genus *Escherichia/Shigella*, a member of the family *Enterobacteriaceae*, was increased in infants with eczema. The most abundant OTU belonging to genus *Escherichia/Shigella* occupied 8.8% and 8.4% of all OTUs in infants with and without eczema, respectively, and these were enriched in infants with eczema (q-value = 1.98E-152).

The second most abundant OTU and two other abundant OTUs all belonging to the species *Faecalibacterium prausnitzii* accounted for 11.78% of eczematous infants compared with 5.70% of healthy infants (Table 3). The results showed that the species *Faecalibacterium prausnitzii* was significantly enriched in eczematous infants (q-value<1E-160), consistent with an earlier report [27].

Two species that belonged to the genus *Bifidobacterium* were enriched in healthy infants, *B*. *bifidum* and *B*. *longum* (Table 3), which have been proven to reduce the risk of eczema in infants [28, 29]; another species, *B*. *dentium*, was enriched in eczematous infants.

In the genus *Bacteroides*, two species were enriched in eczematous infants while *Bacteroides fragilis* were enriched in healthy infants (Table 3). It was reported that *B*. *fragilis* in infants increased from the age of one month to one year [30], and the bacterial polysaccharide (PSA) produced by *B*. *fragilis* could correct systemic T cell deficiencies and mediate establishment of a proper Th1/Th2 balance for the host [31–35]. Because an overactive Th2 response has been implicated in asthma and allergy, the low proportion of *B*. *fragilis* in eczematous infants (2.28% compared to 5.01% in healthy infants) might indicate an imbalanced Th1/Th2 and partly contribute to allergy-like eczema.

Out of 129 OTUs that were found to belong to the phylum *Verrucomicrobia*, 128 were classified into the genus *Akkermansia*, which accounted for 1.78% of gut microbiota in eczematous infants but only 0.92% in healthy infants. Two abundant OTUs of the genus *Akkermansia* were identified as belonging to the species *Akkermansia muciniphila*, which occupied 94.70% of the genus *Akkermansia* in eczematous infants. In the animal model, higher abundance of *A*. *muciniphila* was associated with allergic dermatitis, wherein *A*. *muciniphila*, being a mucin-degrading bacterium in the intestine, reduced the integrity of intestinal barrier function causing increased uptake of allergenic proteins [36]. Thus, we postulate that higher concentration of *A*. *muciniphila* also contribute to eczema in infants.

Species of the genus *Lactobacillus* are well known as probiotics. For the components of 16S rRNA gene, the region V3/V4 was not suitable for identifying *Lactobacillus* species, so no *Lactobacillus sp*. was identified in the gut microbiota at the species level. However, at the genus level, we found that *Lactobacillus* was indeed increased in healthy infants. One member of the genus *Streptococcus*, *S*. *salivarius*, was enriched in healthy infants (1.88%), emphasizing its anti-inflammatory property [37].

*Ruminococcus gnavus* was more abundant in eczematous infants (Table 3), comprising 3.01% compared to 2.78% in healthy infants. This species was identified and characterized as the predominant dysbiosis in Crohn’s disease (CD) patients compared with unaffected relatives and healthy individuals [38]. This was attributed to the high beta-glucuronidase activity expressed by *R*. *gnavus* [39], which could induce local inflammation [38]. The presence of *R*. *gnavus* is also associated with colonization by *Clostridium difficile*, a major enteric pathogen responsible for antibiotic-associated diarrhea [40].

The mode of delivery and type of feeding are key factors that shape the developing infant microbiota [41, 42]. Although our results revealed that four genera abundant in eczematous infants and three genera abundant in healthy infants were not associated with mode of delivery or type of feeding, the abundance of several species was indeed affected. *Bifidobacterium bifidum* and *Bifidobacterium longum* have been shown to represent part of the dominant bacterial members of the gut microbiota of breast-fed infants [43]. In healthy infants, those two species were dominant in breast-fed infants; but in eczematous infants, only *B*. *longum* was dominant in breast-fed infants. In exclusively formula-fed infants, pathogenic *Escherichia coli* is found at a higher proportion than in breast-fed infants [41]. Although the most abundant OTU was classified to the genus *Escherichia/Shigella* and was abundant in eczematous infants no matter what type of feeding/delivery, in healthy infants, it was increased in caesarean delivery and decreased in natural delivery.

## Conclusions

Our study suggests that the taxa abundance, not the taxonomic composition, may distinguish gut microbiota of healthy infants from those of eczematous infants. The observed differences in genera and species between cases and controls may provide insight into the link between the microbiome and eczema risk.

## Supporting Information

S1 Fig 16S rRNA gene library Shannon diversity curves from two groups. (TIF)

S2 Fig UPGMA trees of all samples showed separation by two groups. (TIF)

S3 Fig Principal coordinate analysis (PCoA) diagram based on weighted UniFrac distance showing the similarity relationships among the 101 samples. (TIF)

S4 Fig Venn chart showing the unique and shared OTUs from two groups. (TIF)

S5 Fig 16S rRNA gene library rank abundance curves from two groups. (TIF)

S1 Table Species richness and diversity estimates of all samples obtained at genetic distances of 3%. (XLS)

S2 Table Abundant families in the gut of infants with and without eczema. (XLS)

S3 Table Abundant genera in the gut of infants with and without eczema. (XLS)
