# Supplementary figures and images for: Altered Gut Microbiota Composition Associated with Eczema in Infants
Source: PLoS One. 2016 Nov 3;11(11):e0166026. doi: 10.1371/journal.pone.0166026 (PMC5094743; doi:10.1371/journal.pone.0166026)

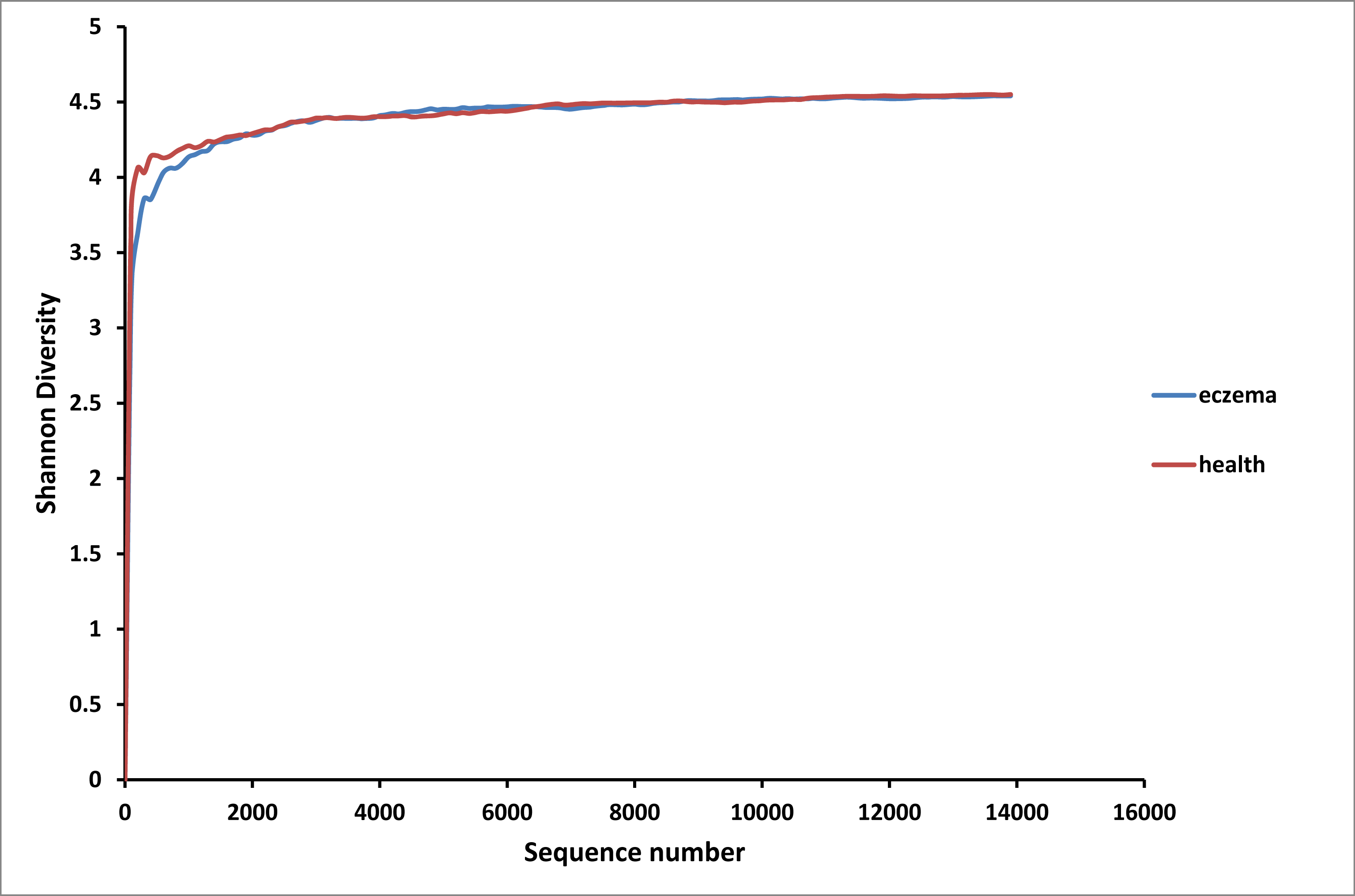

Supplement: S1 Fig — (TIF) [file pone.0166026.s001.tif]

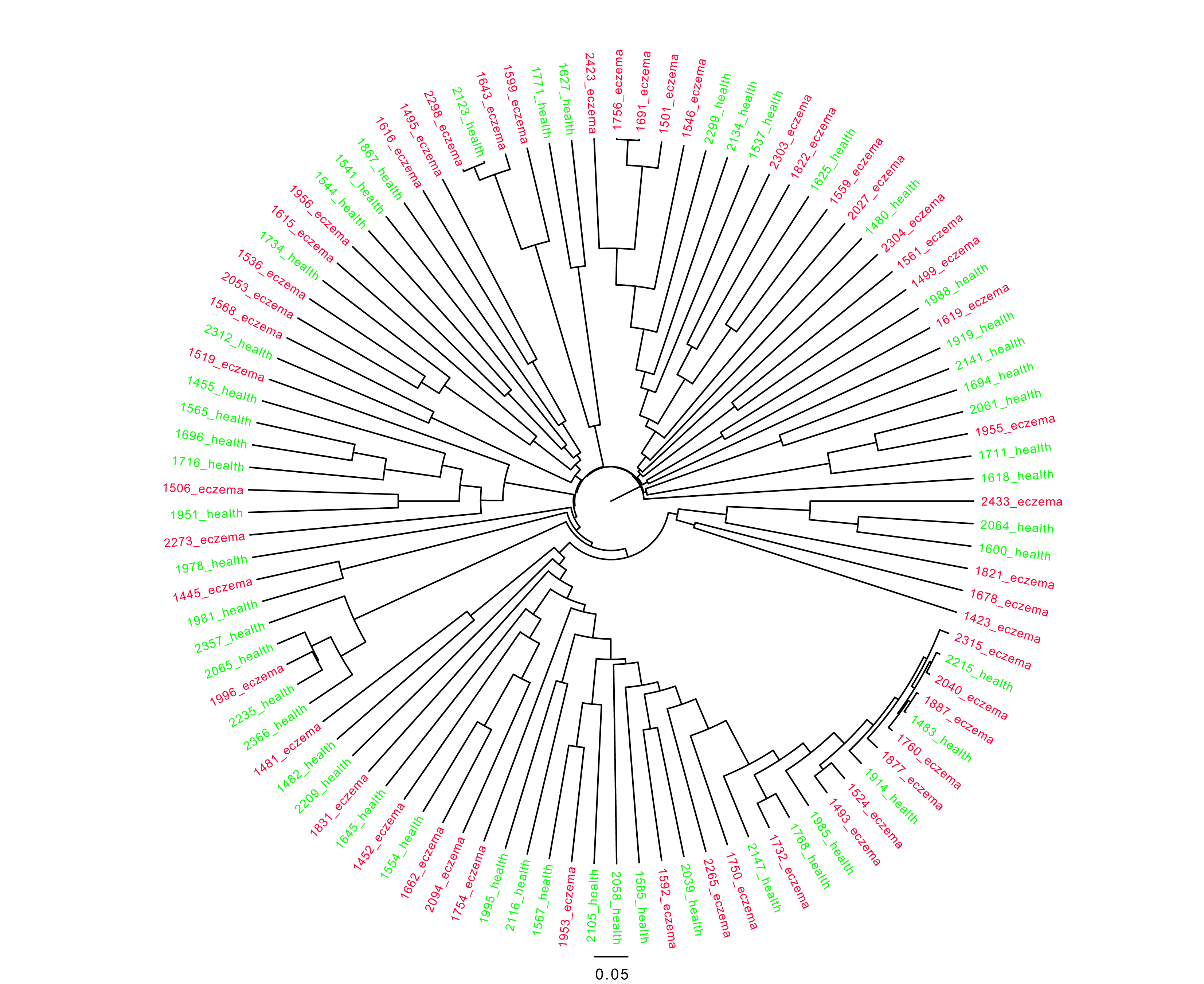

Supplement: S2 Fig — (TIF) [file pone.0166026.s002.tif]

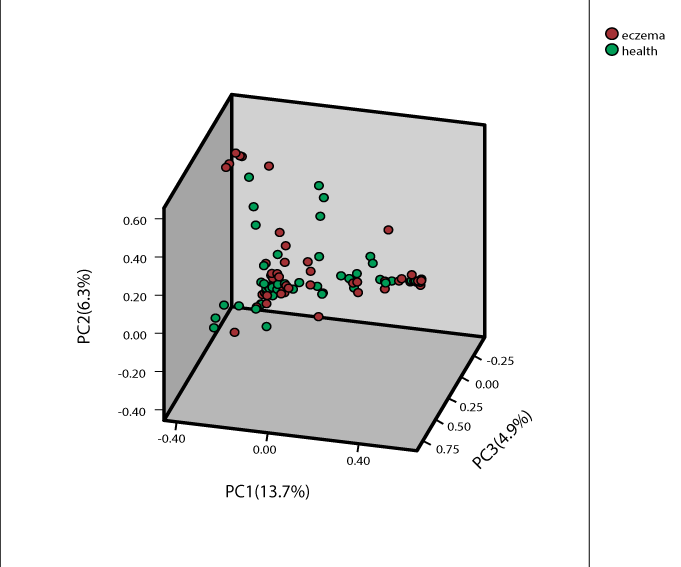

Supplement: S3 Fig — (TIF) [file pone.0166026.s003.tif]

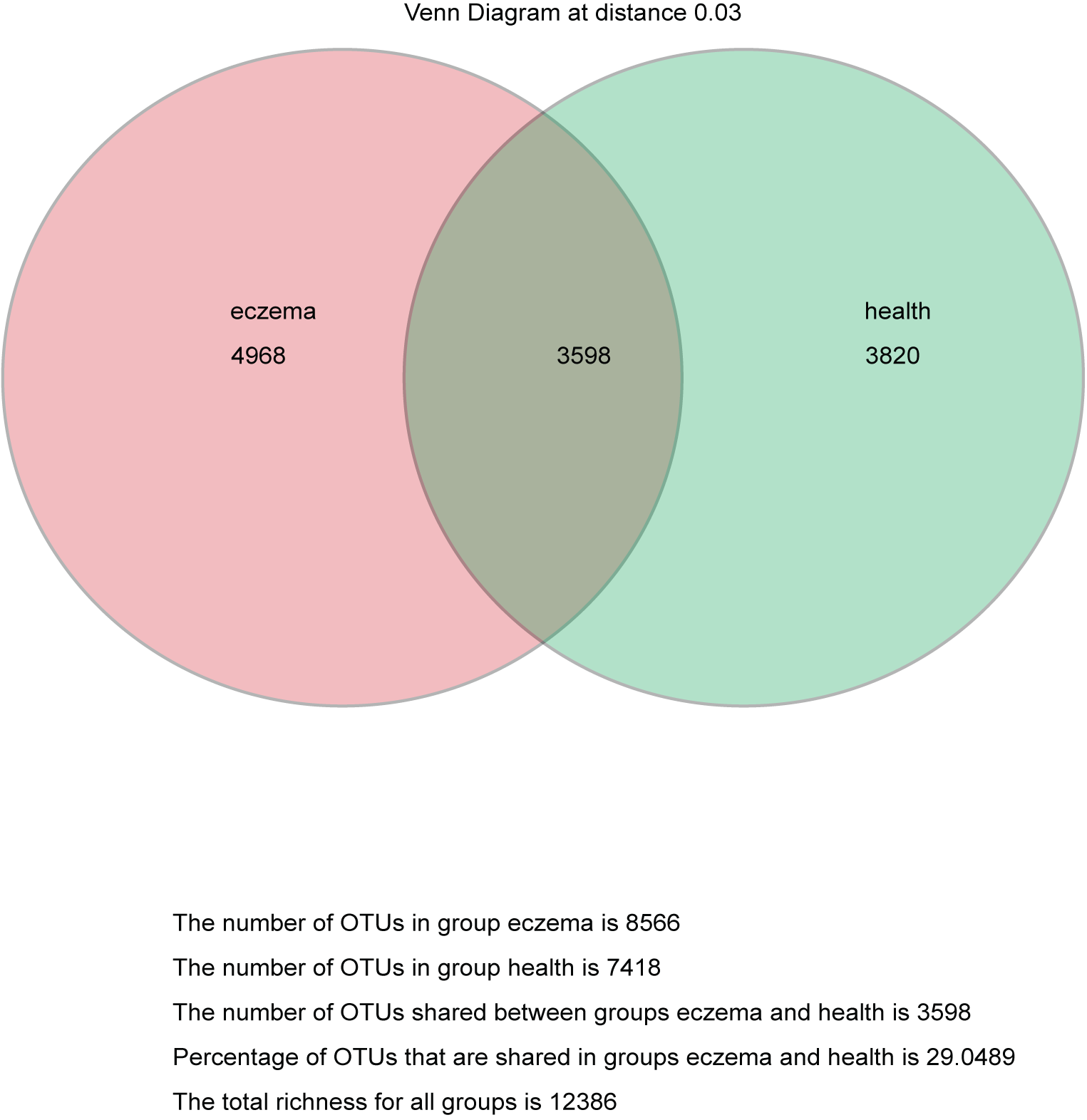

Supplement: S4 Fig — (TIF) [file pone.0166026.s004.tif]

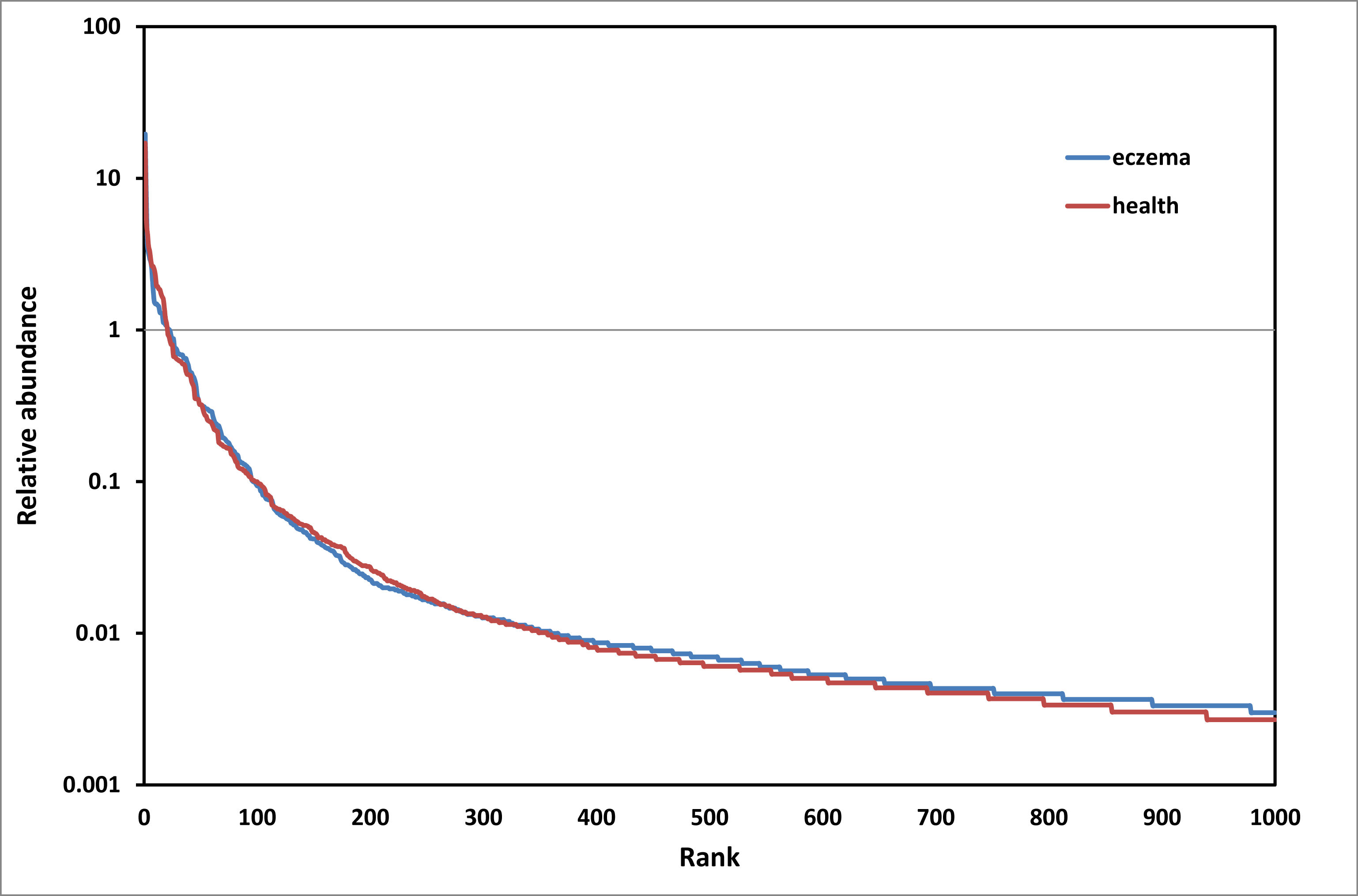

Supplement: S5 Fig — (TIF) [file pone.0166026.s005.tif]
